# Development of a Simple Clinical Risk Score for Early Prediction of Severe Dengue in Adult Patients

**DOI:** 10.1371/journal.pone.0154772

**Published:** 2016-05-03

**Authors:** Ing-Kit Lee, Jien-Wei Liu, Yen-Hsu Chen, Yi-Chun Chen, Ching-Yen Tsai, Shi-Yu Huang, Chun-Yu Lin, Chung-Hao Huang

**Affiliations:** 1 Division of Infectious Diseases, Department of Internal Medicine, Kaohsiung Chang Gung Memorial Hospital, Kaohsiung, 833, Taiwan; 2 Chang Gung University College of Medicine, Tao-Yuan, 333, Taiwan; 3 Division of Infectious Diseases, Department of Internal Medicine, Kaohsiung Medical University Hospital, Kaohsiung, 833, Taiwan; 4 Kaohsiung Medical University, Kaohsiung, 833, Taiwan; 5 Department of Emergency Medicine, Kaohsiung Chang Gung Memorial Hospital, Kaohsiung, 833, Taiwan; Institute of Tropical Medicine (NEKKEN), Nagasaki University, JAPAN

## Abstract

We aimed to develop and validate a risk score to aid in the early identification of laboratory-confirmed dengue patients at high risk of severe dengue (SD) (i.e. severe plasma leakage with shock or respiratory distress, or severe bleeding or organ impairment). We retrospectively analyzed data of 1184 non-SD patients at hospital presentation and 69 SD patients before SD onset. We fit a logistic regression model using 85% of the population and converted the model coefficients to a numeric risk score. Subsequently, we validated the score using the remaining 15% of patients. Using the derivation cohort, two scoring algorithms for predicting SD were developed: models 1 (dengue illness ≤4 days) and 2 (dengue illness >4 days). In model 1, we identified four variables: age ≥65 years, minor gastrointestinal bleeding, leukocytosis, and platelet count ≥100×10^9^ cells/L. Model 1 (ranging from −2 to +6 points) showed good discrimination between SD and non-SD, with an area under the receiver operating characteristic curve (AUC) of 0.848 (95% confidence interval [CI], 0.771–0.924). The optimal cutoff value for model 1 was 1 point, with a sensitivity and specificity for predicting SD of 70.3% and 90.6%, respectively. In model 2 (ranging from 0 to +3 points), significant predictors were age ≥65 years and leukocytosis. Model 2 showed an AUC of 0.859 (95% CI, 0.756–0.963), with an optimal cutoff value of 1 point (sensitivity, 80.3%; specificity, 85.8%). The median interval from hospital presentation to SD was 1 day. This finding underscores the importance of close monitoring, timely resuscitation of shock including intravenous fluid adjustment and early correction of dengue-related complications to prevent the progressive dengue severity. In the validation data, AUCs of 0.904 (95% CI, 0.825–0.983) and 0.917 (95% CI, 0.833–1.0) in models 1 and 2, respectively, were achieved. The observed SD rates (in both cohorts) were <3% for patients with a score <1 point, but >50% for those with a score of ≥2 points, irrespective of the day of illness onset, suggesting that our simple risk score can be easily implemented in resource-limited countries for early prediction of dengue patients at risk of SD provided that they have rapid dengue confirmed tests. For patients with other acute febrile illnesses or bacterial infections usually have SD risk score of >1. Thus, these scoring algorithms cannot totally replace good clinical judgement of the physician, and most importantly, early differentiating dengue from other febrile illnesses is critical for appropriate monitoring and management.

## Introduction

Dengue is the most common mosquito-borne arboviral disease [1]. The World Health Organization (WHO) estimates that 2.5 billion people in tropical and subtropical regions worldwide are at risk [2]. In Taiwan, cyclical dengue epidemics have occurred since the 1980s, leading to large disease and economic burdens [3–6]. A wide spectrum of dengue manifestations is seen, ranging from self-limiting fever to death [1, 2]. Currently, there is no licensed vaccine or anti-viral drug against dengue. The cornerstone of management and prevention of dengue-related mortality is early recognition of severe-form dengue requiring intervention [2]. Unfortunately, the appropriate management may be delayed due to the lack of an accurate means to identify patients at risk of developing severe complications early.

The WHO has published two sets of guidelines for dengue severity classification: the 1997 and 2009 guidelines [2, 7]. In the 1997 guidelines, dengue severity was categorized into mild self-limiting illness, dengue fever (DF), dengue hemorrhagic fever (DHF), and dengue shock syndrome (DSS), the most severe form [7]. However, this classification may not be universally applicable, as severe clinical features also occur in patients not meeting the criteria for DHF/DSS [8, 9]. Consequently, the 2009 WHO dengue guidelines classified dengue into dengue with and without warning signs, and severe dengue (SD) [2]. The proposed warning signs include abdominal pain or tenderness, persistent vomiting, clinical fluid accumulation, mucosal bleed, lethargy or restlessness, liver enlargement >2 cm, and an increase in the hematocrit concurrent with a rapid decrease in the platelet count [2]. However, the sensitivity of each individual warning sign in predicting subsequent SD is reportedly extremely low [10]. Moreover, in addition to these proposed warning signs, gastrointestinal bleeding and leukocytosis are found in the majority of fatal dengue cases [11–13], emphasizing the importance of continuous analyses of the relevant findings to assist clinicians in distinguishing SD from non-SD at the early disease stages.

In this study, a retrospective review was conducted using data collected from adult dengue patients before the development of SD at two medical centers in Taiwan, with the purposes of identifying a means to allow early identification of patients at high risk of progression to SD and to facilitate timely intervention. In particular, we aimed to develop a scoring system that can be easily and accurately applied clinically to identify patients at greater risk for SD upon arrival.

## Materials and Methods

### Ethics statement

The study was reviewed and approved by the Institutional Review Board of Kaohsiung Chang Gung Memorial Hospital (KSCGMH) and Kaohsiung Medical University Hospital (KMUH) (Document no. 104-2150B). Informed consent was not obtained, as the data were anonymized and de-identified prior to analysis.

### Patients and setting

This retrospective study was performed by extracting data from dengue patients managed at KSCGMH (2,500 beds), between July 1, 2002 and May 31, 2015, and KMUH (1,700 beds), during 2009–2011. These hospitals serve as primary care and tertiary referral centers in Taiwan. The study inclusion criteria were adult patients (≥18 years) with laboratory-confirmed dengue virus (DENV) infection. Children (<18 years old) and cases of SD at the time of hospital presentation were excluded from analysis. All dengue cases included in this study were confirmed by at least one of the following criteria: (i) positive DENV-specific real-time reverse transcription polymerase chain reaction (RT-PCR; QuantiTect SYBR Green RT-PCR kit; Qiagen, Hilden, Germany), (ii) a fourfold increase in DENV-specific immunoglobulin G (IgG) antibody in the convalescent serum compared to in the acute-phase serum, and/or (iii) detection of DENV-specific nonstructural glycoprotein-1 antigen (Bio-Rad Laboratories, Marnes-la-Coquette, France) in the acute-phase serum [14, 15]. All diagnostic tests were performed by the Center for Disease Control, Taiwan.

### Case classification

We utilized both the 1997 and 2009 WHO guidelines for defining disease severity [2, 7]. The diagnosis of DHF was established based on the presence of fever, hemorrhage, thrombocytopenia (platelet count <100×10^9^ cells/L), and clinical evidence of plasma leakage (presence of hemoconcentration, pleural effusion, ascites, and/or hypoalbuminemia). DHF grades 1 and 2 were defined as a positive tourniquet test result being the only hemorrhagic manifestation and the occurrence of spontaneous bleeding such as mucosal or gastrointestinal bleeding, respectively. DHF grades 3 and 4 were grouped as DSS, defined as cases of DHF with circulatory failure manifested by a rapid, weak, and narrowing pulse (<20 mmHg), or the presence of profound shock (systolic blood pressure <90 mmHg) [7]. With respect to the 2009 WHO dengue definitions, cases were categorized as non-SD and SD. SD was defined as cases with severe plasma leakage (hematocrit change >20%) with shock (systolic blood pressure <90 mmHg) or fluid accumulation with respiratory distress, or severe bleeding or organ impairment [2].

### Data extraction

Because 49.7% of dengue cases were enrolled during 2002–2008, prior to the introduction of the 2009 revised classification [2], a standardized form for clinical data collection was designed to classify laboratory-confirmed dengue patients according to both the 1997 and 2009 WHO dengue definitions [2, 7]. The data were mainly retrieved from the hospital electronic medical records, and were supplemented by a secondary manual search. Data collected included demographic characteristics, reported morbidity, and the presence or absence of signs/symptoms, as well as the results of laboratory tests and radiography/ultrasound examinations at the time of hospital presentation and during the entire clinical course, and thereby determined the level of severity according to each of the classifications [2, 7]. The exact day the patients fulfilled the criteria for DSS and SD was also determined. Data regarding the number of days from (i) the onset of dengue illness to hospital presentation, (ii) the onset of dengue illness to SD, (iii) the onset of dengue illness to DSS, (iv) the hospital presentation to SD, and (v) the hospital presentation to DSS were recorded.

### Definitions

Elderly patients referred to patients aged ≥65 years [16]. Lethargy and hepatomegaly as warning signs were not included in our analysis owing to lacking information. The warning sign of increased hematocrit concurrent with reduced platelet count was defined as an increase in the hematocrit level >20% concurrent with a drop in the platelet count compared to that at hospital presentation within the first 24–36 h. Clinical fluid accumulation was defined as detection of pleural effusion or ascites using chest radiography and ultrasonography. Hemoconcentration referred to >20% increase in hematocrit, calculated as: (maximum hematocrit − minimum hematocrit) × 100/minimum hematocrit. Leukocytosis was defined as a peripheral white blood cell count (WBC) >10×10^9^ cells/L, and leukopenia as a peripheral WBC <3.0×10^9^ cell/L (reference value, 3.0–10×10^9^ cells/L) [17]. Severe hepatitis was defined as serum alanine aminotransferase and/or aspartate aminotransferase levels >1000 U/L (reference value, 40 U/L) [18]. Minor gastrointestinal bleeding was defined as passage of black tarry stools that did not affect the hemodynamic status and hemoglobin value [19]. Severe gastrointestinal bleeding was defined as hematemesis, hematochezia, or melena coupled with hemodynamic instability (systolic blood pressure <90 mm Hg) and/or a rapid drop in hemoglobin (hemoglobin level <10 g/dL or less) within 48 h [11, 19]. Acute kidney injury was defined as a rapid increase in the serum creatinine level >0.5 mg/dL compared to that at hospital presentation [20]. Rhabdomyolysis was defined as a five-fold increase in the serum concentrations of creatine phosphokinase above the upper limit of the normal range (reference value, 13–130 U/L), with a >95% creatine phosphokinase-muscle fraction and detection of myoglobin levels in serum or urine. [21–23]. Hemophagocytosis was defined as fever, splenomegaly, pancytopenia, hypertriglyceridemia, hyperferritinemia, and demonstration of hemophagocytosis in the peripheral blood or bone marrow [24]. Severe organ involvement included impaired consciousness, severe hepatitis, acute kidney injury, acute respiratory failure, rhabdomyolysis, disseminated intravascular coagulopathy, and hemophagocytosis [2, 24].

### Statistical analysis

**Fig 1:**
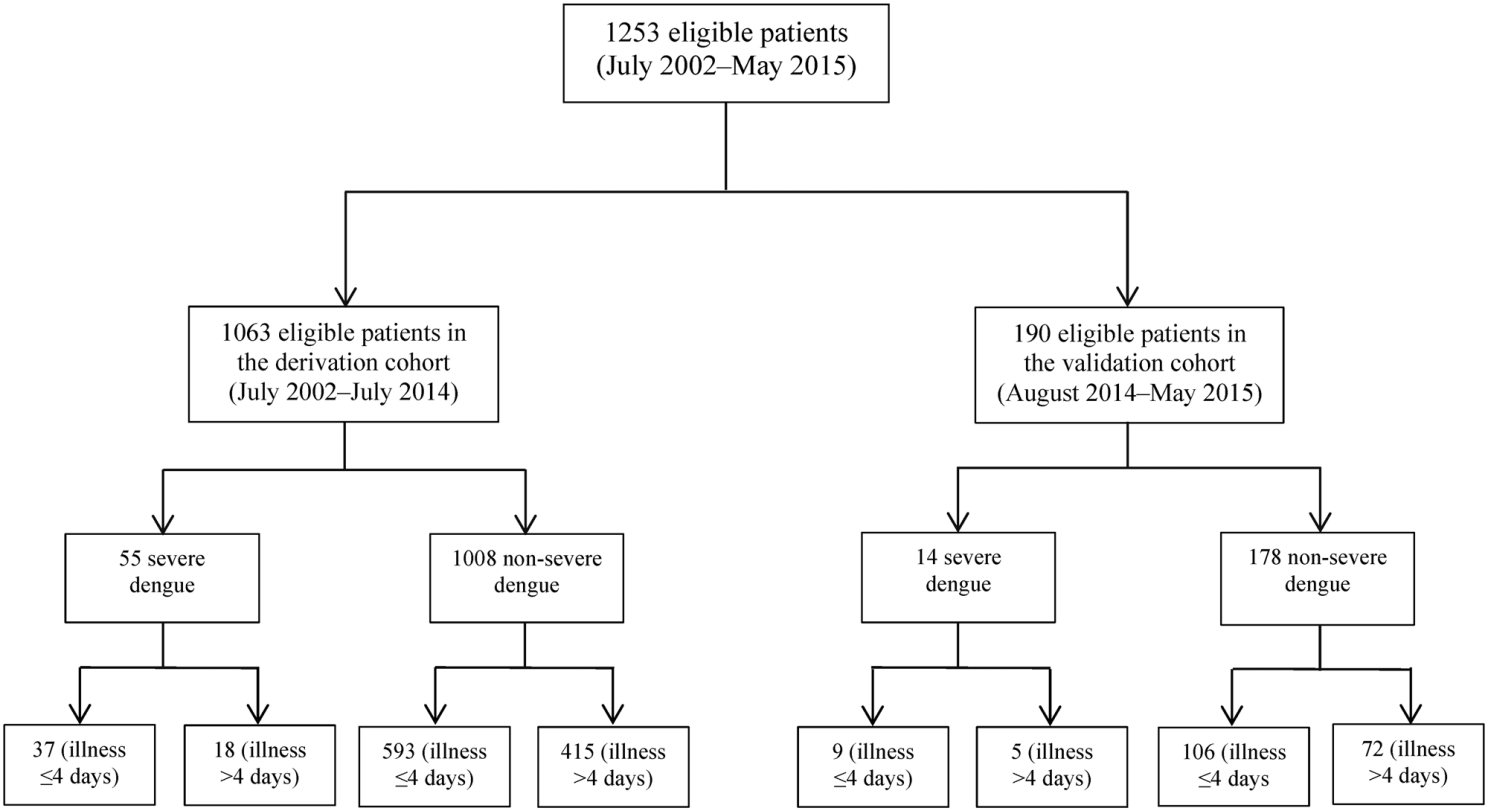
The study flow-chart.

A study flow-chart is shown in Fig 1. We split the study population into two groups according to the study period: one for model derivation (between July 1, 2002 and July 31, 2014) (85% of the population) and the other for model validation (August 1, 2014 to May 31, 2015) (the remaining 15%). In both the derivation and validation cohorts, patients were separated into subgroups according to the days of dengue illness, as defined by WHO 2009 [2]: (i) patients with dengue illness ≤4 days (febrile phase), and (ii) those with dengue illness lasting >4 days (defervescence phase). To disclose the early predictors for SD, the clinical information at the time of hospital presentation before SD onset was analyzed. In the derivation cohort, demographic, clinical symptom/signs, and laboratory data of patients with SD and non-SD were compared. Mann-Whitney U tests and chi-square or Fisher’s exact tests were used to determine statistical significance for continuous and categorical variables, respectively. Differences were considered significant at P ≤0.05. Significant variables in the univariate analyses were entered into a multivariate logistic regression model (Forward Wald) to identify independent predictors of SD and to estimate their relative regression coefficients. We converted the coefficients for the independent predictors into a simplified risk score system, as previously reported [25]. Specifically, we calculated the number of points assigned to each independent predictor by dividing its regression coefficient by the smallest coefficient in the model and rounded this quotient to the nearest whole number. We calculated each patient’s risk score by summing up the points of all variables present on admission, and assessed the predictive score model discrimination using receiver operating characteristic curve (ROC) analysis and by measuring the area under the curve (AUC), which defines how well the model can discriminate between patients with SD and non-SD. AUCs between 0.90–1, 0.80–0.90, 0.70–0.80, 0.60–0.70, and 0.50–0.60 were defined as excellent, good, fair, poor, and fail, respectively [26]. The optimal cutoff value was obtained by ROC curve analysis, and its sensitivity and specificity for predicting SD were measured. The Cochran-Armitage test was used to assess the dose-gradient relationship between the corresponding risk scores and observed rates of SD. Subsequently, the prediction score was applied to the validation cohort, where the discrimination ability of the score was again assessed by analysis of the AUC of ROC. SPSS statistical software (version 17.0; SPSS Inc., Chicago, IL) was used for all data analyses.

## Results

### Study population

A total of 1253 patients (1063 patients in the derivation cohort and 190 in the validation cohort) with laboratory-confirmed DENV infection were analyzed. Of these, 1051 (83.9%) patients were treated in KSCGMH, and 202 (16.1%) in KMUH. None of the females were pregnant. In the derivation cohort, there were 55 SD patients (32 men and 23 women; median age, 66 years), and 1008 (464 men and 544 women; median age, 51 years) non-SD patients, based on the WHO 2009 definitions. Of the 55 SD patients, 5 (9%), 27 (49%), and 23 (41.8%) were classified—according to the WHO 1997 criteria—as having DF, grades 1–2 DHF, and DSS, respectively; among the 1008 non-SD patients, 99 (9.8%) were classified as grades 1–2 DHF and 909 (90.2%) as DF. A total of 190 patients (99 men and 91 women; median age, 54 years) were analyzed in the validation cohort. In the validation cohort, with WHO 1997 classification, 167 (87.9%) had DF, 20 (10.5%) DHF and 3 (1.6%) DSS; with WHO 2009 classification, 176 (92.6%) had non-SD and 14 (7.4%) SD. Among the total 69 SD patients (26 of them were DSS), 63 patients was managed in KSCGMH, and 6 in KMUH; of these, 30 (43.4%) patients were enrolled in 2002, 6 (8.7%) in 2006, 4 (5.8%) in 2009, 5 (7.2%) in 2010, 6 (8.6%) in 2011, 2 (2.9%) in 2012, 15 (21.7%) in 2014 and 1 (1.4%) in 2015. The demographic and clinical features of the included patients are shown in Table 1.

**Table 1:** Characteristics and outcomes for dengue patients in the derivation and the validation cohorts.

Variable	Derivation cohort(n = 1063)	Validation cohort (n = 190)
Median age (range), years	51 (18–91)	54 (18–93)
Age ≥65 years, no. (%)	194 (18.3)	56 (29.4)
Male, no. (%)	496 (46.6)	99 (52.1)
Comorbid condition, no. (%)		
Type 2 DM only	54 (5.1)	7 (3.7)
Hypertension only	118 (11.1)	33 (17.3)
Type 2 DM and hypertension	70 (6.6)	8 (4.2)
Type 2 DM, hypertension, and others	22* (2.1)	8^†^ (4.2)
Hypertension and chronic kidneydisease	4 (0.4)	5 (2.6)
Chronic kidney disease only	5 (0.5)	2 (1.0)
G6PD deficiency only	2^‡^ (0.2)	0
2009 WHO dengue classification, no. (%)		
Non-severe dengue	1008 (94.2)	176 (92.6)
Severe dengue	55 (5.1)	14 (7.4)
1997 WHO dengue classification, no. (%)		
Dengue fever	914 (85.9)	167 (87.9)
Grades 1 and 2 DHF	126 (11.8)	20 (10.5)
DSS	23 (2.2)	3 (1.6)
Serotype, no./No. (%)		
DENV 1	17/738 (2.3)	42/63 (66.7)
DENV 2	639/738 (87)	19/63 (30.2)
DENV 3	81/738 (11)	2/63 (3.1)
DENV 4	1/738 (0.1)	0
Median time from illness onset to hospital presentation (range), days	4 (1–15)	4 (1–13)
Fatal^§^, no. (%)	12 (1.1)	3 (1.6)

DM = diabetes mellitus; DENV = dengue virus; DHF = dengue hemorrhagic fever; DSS = dengue shock syndrome; G6PD = glucose-6-phosphate dehydrogenase; no./No. = number of cases/number of overall cases with data available for evaluation; WHO = World Health Organization.

*Among the 22 DM patients with hypertension, previous stroke was noted in 10 patients, ischemic heart disease in 6, chronic kidney disease in 5, and chronic kidney disease with previous stroke in one.

^†^Among the 8 DM patients with hypertension, ischemic heart disease was noted in 5 patients and chronic kidney disease in 3.

^‡^One of them experienced acute hemolysis anemia during the dengue illness.

^§^Of these15 fatal patients, refractory shock due to severe plasma leakage with multiple organs failure was found in 6 patients, intractable massive gastrointestinal bleeding with hypovolemic shock and multiple organs failure in 4, bacteremia with septic shock in 4 (one had concurrent candidemia), and rhabdomyolysis and nosocomial pneumonia in one.

### Characteristics of the 69 SD patients in the derivation and the validation cohorts (Table 2)

**Table 2:** Characteristics of the 69 severe dengue patients in the derivation and the validation cohorts*.

Variable	Severe dengue(n = 69)
**Demographics characteristics**	
Median age (range), years	66 (25–85)
Age ≥65 years, no. (%)	45 (65.2)
Male, no. (%)	43 (62.3)
Comorbid condition, no. (%)	
Type 2 DM only	2 (2.8)
Hypertension only	13 (18.8)
Type 2 DM and hypertension	12 (17.4)
Type 2 DM, hypertension, and others	7^†^ (10.1)
Hypertension and chronic kidney disease	5 (7.2)
Chronic kidney disease only	2 (2.8)
G6PD deficiency only	1 (1.4)
DENV serotype, no./No. (%)	
DENV 1	5/39 (12.8)
DENV 2	32/39 (82.1)
DENV 3	2/39 (5.1)
DENV 4	0
1997 WHO dengue classification, no. (%)	
Dengue fever	12 (17.4)
Grades 1 and 2 DHF	31 (45)
DSS	26 (37.6)
Median time from illness onset to hospital presentation (range), days	3 (1–7)
Median time from illness onset to severe dengue (range), days	5 (2–10)
Median time from hospital presentation to severe dengue (range), days	1 (1–5)
Median time from illness onset to DSS (range), days (no.)	6 (2–10) (n = 26)
Median time from hospital presentation to DSS (range), days (no.)	1 (1–5) (n = 26)
**Complication during the entire clinical course and outcome**^‡^**, no. (%)**	
Hemoconcentration (increase in Hct >20%)	48 (69.5)
Gastrointestinal bleeding (included minor and severe bleeding)	40 (57.9)
Acute kidney injury	34 (49.3)
Severe gastrointestinal bleeding	25 (36.2)
Acute respiratory failure	22 (31.9)
Impaired consciousness	20 (29)
Severe hepatitis (ALT and/or AST >1000 U/L)	14 (20.3)
Rhabdomyolysis	7 (10.1)
Bacteremia	5 (7.2)
Pneumonia	4 (5.8)
Hemophagocytosis	2 (2.8)
Disseminated intravascular coagulopathy	2 (2.8)
Acute hemolytic anemia	1^§^ (1.4)
Candidemia	1 (1.4)
Fatal, no. (%)	15 (21.7)

ALT = alanine aminotransferase; AST = aspartate aminotransferase; DM = diabetes mellitus; DENV = dengue virus; DHF = dengue hemorrhagic fever; DSS = dengue shock syndrome; G6PD = glucose-6-phosphate dehydrogenase; Hct = hematocrit; no./No. = number of cases/number of overall cases with data available for evaluation; WHO = World Health Organization.

*Based on 2009 WHO dengue classification scheme.

^†^Among the 7 DM patients with hypertension, chronic kidney disease was noted in 4 patients, previous stroke in 2, and chronic kidney disease with previous stroke in one.

^‡^One patient might had more than one complication.

^§^The patient had underlying G6PD deficiency.

The median length from the onset of dengue illness to hospital presentation of the 69 SD patients was 3 (range, 1–7) days. Of the 69 SD patients, 49 (71%) patients presented to the hospital between days 1 and 4 after the onset of illness. Among the 69 SD patients, the three most common warning signs at presentation were abdominal pain (45%), vomiting (35%), and presence of pleural effusion (31.2%). Minor gastrointestinal bleeding was noted in 13 (19%) patients at the time of hospital presentation. The median hematocrit level and platelet count on arrival were 36.9% (37.3% for males and 36.4% for females; range, 21.9–52.1) and 36 ×10^9^ cells/L (range, 1.5–191), respectively. Leukocytosis was seen in 16 (23.1%) patients upon arrival; of these, 8 (50%) developed severe gastrointestinal bleeding after hospitalization, whereas two (12.5%) patients presented *Moraxella lacunata* and *Enterococcus faecalis* bacteremia upon arrival. The median intervals from illness onset and hospital presentation to SD development were 5 (range, 2–10) and 1 (range, 1–5) days, respectively.

Table 2 shows the complications of the 69 SD patients during the entire clinical course. Compared to non-SD patients, SD patients had a significant increase in length of hospital stay (median [range], 5 [1–24] days vs. 10 [4–80] days; P < 0.001). Hemoconcentration, gastrointestinal bleeding, acute kidney injury, impaired consciousness, severe hepatitis, rhabdomyolysis, hemophagocytosis, disseminated intravascular coagulopathy, and acute hemolytic anemia were found in 48 (69.5%), 40 (57.9%), 34 (49.3%), 20 (29%), 14 (20.3%), 7 (10.1%), 2 (2.8%), 2 (2.8%), and 1 (1.4%) patients, respectively. Of the 40 SD patients with gastrointestinal bleeding, 25 (62.5%) patients experienced severe gastrointestinal hemorrhage during the hospitalization. Notably, transfusion of platelets and/or other blood component(s) (i.e., packed red blood cells and/or fresh frozen plasma) was administered to the patients experiencing severe gastrointestinal bleeding. Among the total 69 SD patients, bacteremia was noted in 5 (7.2%) patients and nosocomial pneumonia in 4 (5.8%). Bacteremia (*Klebsiella pneumoniae* [n = 2], *Moraxella lacunata* [n = 1], and *Enterococcus faecalis* [n = 1]) was detected upon arrival in 4 patients (2 with leukopenia and 2 with leukocytosis), while one patient with diabetes developed *Acinetobacter baumannii* bacteremia concurrent with candidemia due to a nosocomial infection. Twenty two (31.9%) patients experienced respiratory failure requiring mechanical ventilation. DSS occurred in 26 (37.6%) of the 69 SD patients. Of the 26 DSS patients, presence of pleural effusion (46.1%), gastrointestinal bleeding (42.3%), and vomiting (38.5%) were the three most common warning signs on arrival. The median periods between dengue illness onset and hospital presentation to DSS were 6 (range, 2–10) and 1 (range, 1–5) days, respectively. Leukocytosis was found in 4 (15.3%) of the 26 DSS patients upon arrival. Overall, 15 SD patients died (mortality rate, 21.7%).

### Characteristics of the 15 fatal patients

There were 15 fatal dengue cases between the periods of July 1, 2002 to May 31, 2015 (12 in derivation cohort and 3 in validation cohort). All cases were treated in KSCGMH. The median age of the 15 fatal cases was 63 years (range, 33–85), and 12 (80%) were males. Of these, 6 (40%) patients had hypertension, 3 (20%) chronic kidney disease, and 2 (13.3%) diabetes mellitus. The three leading symptoms other than fever at presentation among the 15 deceased cases were cough (60%), bone pain (53.3%) and vomiting (40%). The median time from the onset of illness to hospital presentation was 3 days (range, 1–8). Furthermore, the time lapses from onset of illness to SD ranged from 2 to 10 days (median = 5 days). With regard to the laboratory data at the time of presentation for 15 fatal patients, leukocytosis was seen in 4 (26.7%) patients; the median hematocrit concentration and platelet count were 33.8% (range, 24.5–52.1) and 51 × 10^9^ cells/L (range 2.5–170), respectively. Of the 15 deceased patients, acute kidney injury was found in 13 (86.7%) patients, gastrointestinal bleeding in 12 (80%), bacteremia in 4 (26.75) (*K*. *pneumoniae* in 2, and *E*. *faecalis* and *A*. *baumannii* each in one), severe hepatitis in 2 (13.3%), as well as rhabdomyolysis, disseminated intravascular coagulopathy and candidemia each in one (each 6.7%).

### Analysis of risk factors associated with SD

As shown in Table 3, in the febrile phase (dengue illness ≤4 days), univariate analyses revealed that the following factors were significantly associated with SD: older age, had any comorbidity, presence of minor gastrointestinal bleeding, absence of rash, leukocytosis (WBC >10×10^9^ cells/L), and platelet count <50 × 10^9^ cells/L. Multivariate analysis revealed age ≥65 years (adjusted odds ratio [aOR] 7.502; 95% confidence interval [CI], 3.084–18.251; P <0.001), presence of minor gastrointestinal bleeding (aOR 113.733; 95% CI, 13.910–929.44; P <0.001), leukocytosis (WBC >10×10^9^ cells/L) (aOR 247.668; 95% CI, 28.209–2174.439; P <0.001), and platelet count ≥100 × 10^9^ cells/L (aOR 0.037; 95% CI, 0.002–0.680; P = 0.026) as independent factors that distinguished SD from non-SD (Table 4).

**Table 3:** Univariate analysis of risk factors associated with severe dengue in the derivation cohort*.

Variable	Severe dengue cases	Non-severe dengue cases		Severe dengue cases	Non-severe dengue cases	
	≤4 days after onset of illness (n = 37)	≤4 days after onset of illness (n = 593)	P	>4 days after onset of illness (n = 18)	>4 days after onset of illness (n = 415)	P
**Demographics characteristics**						
Median age (range), years	66 (25–85)	51 (18–91)	<0.001	65 (30–78)	51 (18–84)	<0.001
Age ≥65 years, no. (%)	21 (56.8)	103 (17.4)	<0.001	13 (72.2)	57 (13.7)	<0.001
Male, no. (%)	21 (56.8)	268 (45.2)	0.178	11 (61.1)	196 (47.2)	0.336
Comorbid condition, no. (%)						
Any one condition	19 (51.3)	140 (23.6)	0.001	11 (61.1)	105 (25.3)	0.002
Type 2 DM only	0	34 (5.7)	-	1 (5.6)	19 (12.7)	>0.99
Hypertension only	6 (16.2)	56 (9.4)	>0.99	3 (16.7)	53 (12.8)	>0.99
Type 2 DM and hypertension	5 (13.5)	38 (6.4)	>0.99	5 (27.8)	22 (5.3)	>0.99
Type 2 DM, hypertension, and others	5 (13.5)	9 (1.5)	>0.99	1 (5.5)	7 (1.7)	>0.99
Hypertension and chronic kidney disease	1 (2.7)	1 (0.2)	>0.99	1 (5.5)	1 (0.2)	>0.99
Chronic kidney disease only	1 (2.7)	2 (0.3)	>0.99	0	2 (0.5)	-
G6PD deficiency only	1 (2.7)	0	-	0	1 (0.2)	-
**Warning signs**^†^^‡^						
Abdominal pain, no. (%)	17 (45.9)	120 (20.2)	>0.99	8 (44.4)	89 (21.4)	>0.99
Vomiting, no. (%)	15 (40.5)	173 (29.2)	>0.99	5 (27.8)	121 (28.2)	>0.99
Mucosal bleeding, no. (%)						
Minor gastrointestinal bleeding^§^	7 (18.9)	2 (0.3)	<0.001	4 (22.2)	5 (1.2)	>0.99
Hemoptysis	0	9 (1.5)	-	0	8 (1.9)	-
Gum bleeding	1 (2.7)	39 (6.6)	0.502	1 (5.6)	47 (11.3)	0.707
Clinical fluid accumulation, no./No. (%)						
Pleural effusion	9/34 (26.4)	28/362 (7.7)	>0.99	7/16 (43.7)	17/234 (7.3)	>0.99
Ascites	6/25 (24)	19/233 (8.2)	>0.99	4/11 (36.4)	10/145 (6.9)	>0.99
Rise in hematocrit level >20% concurrent with drop inplatelet count within 24–36 h after hospital presentation, no./No. (%)	4/30 (13.3)	5/275 (1.8)	>0.99	1/12 (8.3)	9/191 (4.7)	>0.99
**Other symptoms/signs**^†^^‡^**, no. (%)**						
Fever	34 (91.9)	563 (78)	>0.99	16 (88.9)	392 (94.4)	>0.99
Orbital pain	2 (5.4)	68 (11.5)	0.415	2 (11.1)	51 (12.3)	>0.99
Bone pain	14 (37.8)	247 (41.7)	0.732	6 (33.3)	189 (45.5)	0.343
Myalgia	15 (40.5)	228 (38.4)	>0.99	12 (66.7)	132 (31.8)	0.004
Headache	12 (32.4)	257 (43.3)	0.232	6 (33.3)	163 (39.3)	0.806
Diarrhea	5 (13.5)	88 (14.8)	>0.99	2 (11.1)	56 (13.5)	>0.99
Petechial	4 (10.8)	108 (18.2)	0.374	5 (27.8)	121 (29.2)	>0.99
Cough	6 (16.2)	151 (25.5)	0.244	6 (33.3)	105 (25.3)	>0.99
Rash	2 (5.4)	192 (32.3)	<0.001	1 (5.6)	183 (44.1)	0.001
**Laboratory features**^‡^						
Leukocytosis (WBC >10 × 10^9^ cells/L), no./No. (%)	9/37 (24.3)	2/571 (0.4)	<0.001	4/18 (22.2)	2/406 (0.4)	<0.001
Increased hematocrit level >20% within 24–36 h afterhospital presentation, no./No. (%)	5/30 (16.7)	6/278 (2.2)	>0.99	1/12 (8.3)	11/192 (5.8)	>0.99
Median hematocrit % (range) (no.)						
Male	37 (25–49) (n = 21)	37.1 (28.5–50.4) (n = 252)	0.442	36.5 (22.3–49.4) (n = 11)	40 (23.7–51.4) (n = 189)	0.055
Female	34.6 (21.9–49.7) (n = 16)	37.3 (21.4–51) (n = 314)	0.151	36.9 (25.3–49.9) (n = 7)	37.8 (23.7–49.8) (n = 213)	0.923
Median platelet count (× 10^9^ cells/L) (range) (no.)	39 (1.5–190) (n = 37)	108 (2–413) (n = 575)	<0.001	22 (3.0–191) (n = 18)	89 (1.0–344) (n = 409)	<0.001
Severity of thrombocytopenia, no./No. (%)						
Platelet count >150 × 10^9^ cells/L	6/37 (16.2)	140/575 (24.3)	Reference	1/18 (5.6)	63/409 (15.4)	Reference
Platelet count 100–149 × 10^9^ cells/L	3/37 (8.1)	192/575 (33.4)	0.159	0/18 (0)	105/409 (25.6)	-
Platelet count 50–99 × 10^9^ cells/L	9/37 (24.3)	138/575 (24)	0.437	4/18 (22.2)	123/409 (30)	0.549
Platelet count <50 × 10^9^ cells/L	19/37 (51.4)	105/575 (18.3)	0.003	13/18 (72.2)	118/409 (28.8)	0.072
Elevated ALT^‖^(normal value < 40 U/L), no./No. (%)	5/24 (20.8)	6/352 (1.7)	>0.99	1/11 (9)	3/217 (1.4)	>0.99
Elevated AST^‖^(normal value < 40 U/L), no./No. (%)	7/28 (25)	8/395 (2)	>0.99	1/10 (10)	10/274 (3.6)	>0.99

ALT = alanine aminotransferase; AST = aspartate aminotransferase; DM = diabetes mellitus; DENV = dengue virus; DHF = dengue hemorrhagic fever; DSS = dengue shock syndrome; G6PD = glucose-6-phosphate dehydrogenase; no./No. = number of cases/number of overall cases with data available for evaluation; WHO = World Health Organization; WBC = white blood cell count.

*Based on 2009 WHO dengue classification scheme.

^†^One patient might had more than one symptom/sign.

^‡^Data were obtained at the time of hospital presentation.

^§^Severe gastrointestinal bleeding developed in 21 of the SD patients during the hospitalization. In contrast, none of the non-SD patients experienced severe gastrointestinal bleeding during the entire clinical course of dengue illness.

^‖^ALT/AST rising to above 400 U/L (≥10 times upper limit of the normal range) [18].

**Table 4:** Multivariable model and risk score for severe dengue according to the days of dengue illness.

	Severe dengue cases	Non-severe dengue cases	Odds ratio	Coefficient	95% CI	P	Risk score weight
**Model 1 (dengue illness ≤4 days)**							
Age							
≥65 years	21	101	7.502	2.015	3.084–18.251	<0.001	1
<65 years	16	474	1				0
Minor gastrointestinal bleeding							
Yes	7	2	113.733	4.734	13.910–929.944	<0.001	2
No	30	573	1				0
Leukocytosis (WBC >10 × 10^9^ cells/L)							
Yes	9	2	247.668	5.512	28.209–2174.439	<0.001	3
No	28	573	1				0
Platelet count ≥100 × 10^9^ cells/L							
Yes	9	332	0.037	–3.296	0.002–0.680	0.026	–2
No	28	243	1				0
**Model 2 (dengue illness >4 days)**							
Age							
≥65 years	13	56	16.183	2.784	4.291–61.037	<0.001	1
<65 years	5	350	1				0
Leukocytosis (WBC >10 × 10^9^ cells/L)							
Yes	4	2	72.919	4.289	8.197–648.707	<0.001	2
No	14	404	1				0

CI = confidence interval; WBC = white blood cell count.

In the defervescence phase (dengue illness >4 days), variables significantly associated with SD in the univariate analyses were older age, had any comorbidity, myalgia, absence of rash, and leukocytosis (WBC >10×10^9^ cells/L) (Table 3). Multiple logistic regression revealed age ≥65 years (aOR 16.183; 95% CI, 4.291–61.0370; P <0.001) and leukocytosis (WBC >10×10^9^ cells/L) (aOR 72.919; 95% CI, 197–648.707; P <0.001) as independent factors that differentiated SD from non-SD (Table 4).

### Risk scoring system

**Fig 2:**
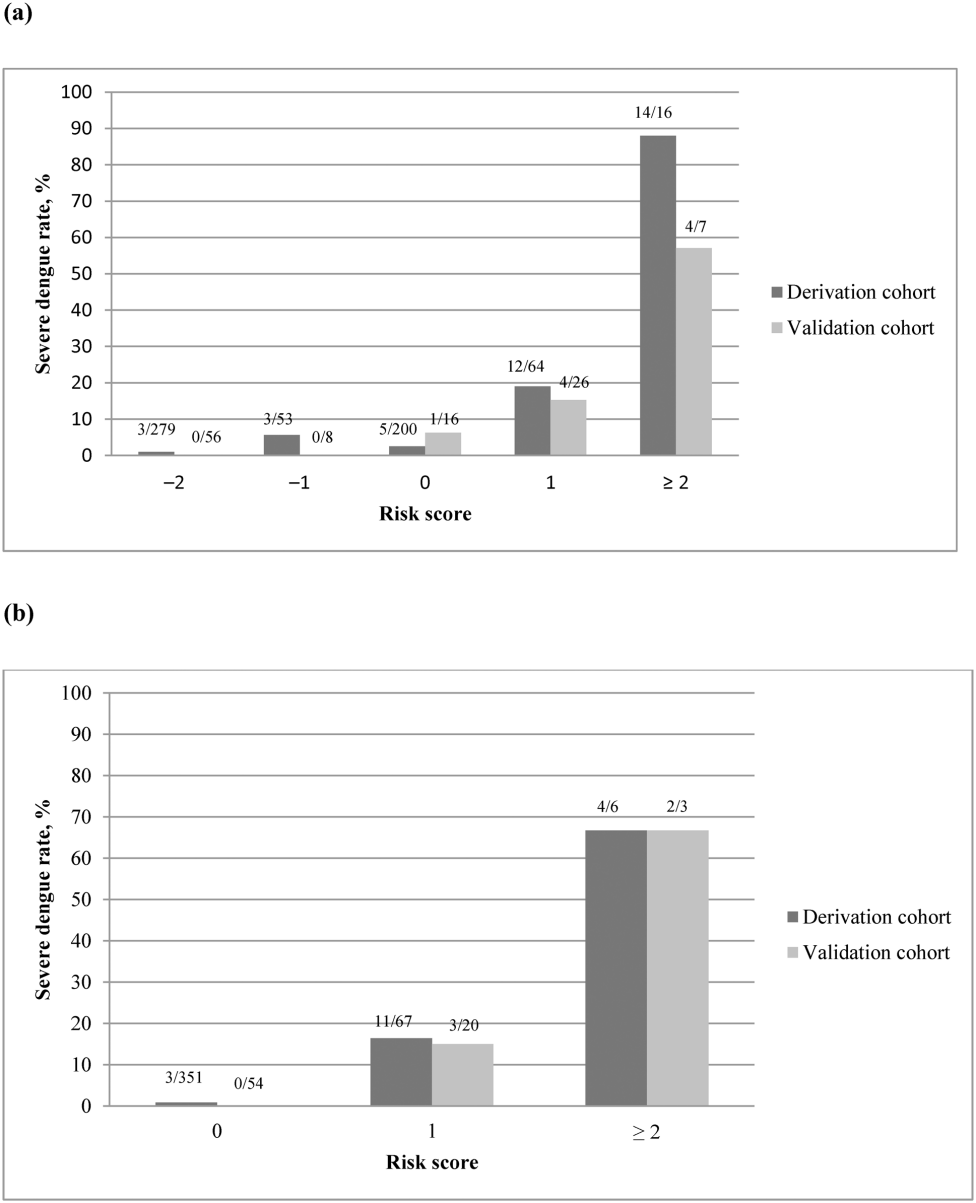
Observed severe dengue rates by risk score strata: (a) patients with dengue illness ≤4 days; (b) patients with dengue illness lasting >4 days. Each box indicates the percentage of severe dengue cases for each corresponding risk score.

**Fig 3:**
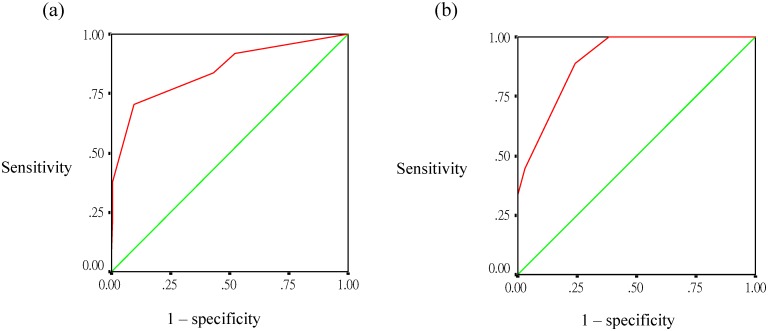
Discriminatory performance of risk score for differentiating severe dengue from non-severe dengue among patients with dengue illness ≤4 days: (a) derivation cohort, (b) validation cohort.

Using standard methods [25], two scoring algorithms were developed based on the above significant predictors for SD for models 1 (dengue illness ≤4 days) and 2 (dengue illness >4 days) (Table 4). For model 1, the SD predictive score included 3, 2, 1, and −2 points for leukocytosis (WBC >10 ×10^9^ cells/L), minor gastrointestinal bleeding, age ≥65 years, and platelet count ≥100 × 10^9^ cells/L, respectively. Hence, model 1 had a maximum of +6 points and a minimum of −2 points. The observed rates of SD for risk scores of –2, –1, 0, 1, and ≥2 points were 1%, 5.6%, 2.5%, 19%, and 88%, respectively (P <0.0001, Cochran-Armitage trending test) (Fig 2a). The SD predictive scoring model developed using these 4 predictors showed good discrimination between SD and non-SD, with an AUC of 0.848 (95% CI, 0.771−0.924, P <0.001) (Fig 3a). The optimal cutoff value of the SD risk score for predicting SD was 1 point, and the sensitivity and specificity of this cutoff were estimated at 70.3% and 90.6%, respectively. Of 26 patients with a score ≥1 point, abdominal pain was found in 15 (57.7%) patients, persistent vomiting in 12 (46.2%), pleural effusion in 8 (30.7%), minor gastrointestinal bleeding in 6 (23%), ascites in 5 (19.2%), increased hematocrit concurrent with reduced platelet count in 3 (11.5%), and gum bleeding in one (3.8%).

**Fig 4:**
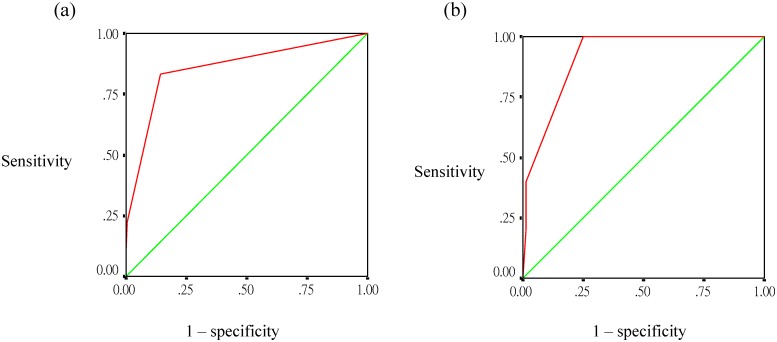
Discriminatory performance of risk score for differentiating severe dengue from non-severe dengue among patients with dengue illness lasting >4 days, (a) derivation cohort, (b) validation cohort.

The score in model 2 included 2 points for leukocytosis (WBC >10×10^9^ cells/L) and 1 point for age ≥65 years, resulting in a maximum score of +3 points and a minimum of 0 points. Among the patients with a score of 0, the observed rate of SD was only 0.85%. In contrast, for those whose scores were 1 and ≥ 2 points, the observed SD rates were 16.4% and 66.7% (P <0.0001, Cochran-Armitage trending test), respectively (Fig 2b). The ROC curve showed a good ability to predict SD, with an AUC of 0.859 (95% CI, 0.756−0.963; P <0.001) (Fig 4a). The sensitivity and specificity using a risk score of 1 point as the cutoff for predicting SD were estimated at 80.3% and 85.8%, respectively. Fifteen patients scored ≥1 point, and among them, abdominal pain and pleural effusion was found in 7 (46.7%) patients each; persistent vomiting, ascites, and minor gastrointestinal bleeding in 4 (26.7%) patients each; and increased hematocrit concurrent with reduced platelet count, as well as gum bleeding, in one (6.7%) patient each.

### Internal validity

There were 190 (176 non-SD and 14 SD) patients in the validation cohort; of these, 115 (106 non-SD and 9 SD) patients presented to the hospital on or before day 4 of illness, while 77 (72 non-SD and 5 SD) patients presented on or after day 5 of illness. Regardless of the day of illness, the observed SD rate increased significantly according to the risk score strata (P <0.0001 by the Cochran-Armitage trending test) (Fig 2a and 2b) when the two proposed scoring systems were applied to patients in the validation cohort. In the validation cohort, model 1 (dengue illness ≤4 days) had an AUC of 0.904 (95% CI, 0.825−0.983; P <0.001) (Fig 3b), with 88.9% sensitivity and 76% specificity using a score of 1 point as the cutoff value. For model 2 (dengue illness >4 days), at a cutoff of 1 point, the score had 100% sensitivity and 76.1% specificity, with an AUC of 0.917 (95% CI, 0.833−1.0; P = 0.002) (Fig 4b).

## Discussion

In our study, the median intervals from illness onset to SD were 5 days. Remarkably, 71% of SD patients in our series presented to the hospital within 4 (median, 3 days; range 1–7) days after the onset of illness, and most patients evolved into SD within a short time frame after admission to a hospital (median, 1 day; range, 1 to 5). Shock and respiratory failure occurred in 37.6% and 31.9% of the SD patients, respectively. Additionally, most of SD cases experienced dengue-related complications such as gastrointestinal bleeding (57.9%), acute kidney injury (49.3%) and severe hepatitis (20.3%). These findings showed that infrequent monitoring of clinical vital parameters (i.e., vital signs and hematocrit level) and suboptimal fluid resuscitation may have contributed to development of dengue-related complications. Timely recognition and prompt aggressive intravenous fluid supplement is the mainstay of treatment for SD [1, 2]. Our findings here indicate that the lack of alertness made clinicians avert from the appropriate fluid resuscitations in some of the SD cases. Our report highlights the urgent need for improving clinicians’ awareness and developing an applicable algorithm that can be easily implemented to identify patients who are at high risk of progressing to SD. Furthermore, the finding of the suboptimal fluid resuscitations in SD cases in this series underscores the importance of a timely effective volume replacement to prevent the progressive dengue severity. Nevertheless, aggressive fluid resuscitation without monitoring may lead to fluid overload―particular in re-absorption phase of dengue illness [1, 2]. The importance of this issue cannot be overemphasized.

Previously, Potts et al. developed two clinical decision tree algorithms using age, WBC, percent neutrophils, percent monocytes, platelet count, hematocrit, and aspartate aminotransferase for early identification of DSS in pediatric patients [27]. In another study of 917 confirmed adult dengue cases, Lee et al. established a decision tree algorithm and found that clinical bleeding, serum urea, and serum total protein were independently associated with DHF [28]; moreover, in a decision tree algorithm designed by Tanner et al., platelet count, viremia, and the presence of pre-existing anti-dengue IgG antibodies were used to predict the occurrence of DHF [29]. However, these studies defined severe dengue illness according to the WHO 1997 criteria, which require the presence of four criteria to classify DHF/DSS, resulting in problems with the classification and detection of severe cases, as not all DHF cases are severe, and not all mild cases are DF [8, 9]. Indeed, our study demonstrated that 9% of SD cases did not fulfill the DHF criteria and were misclassified as DF by the WHO 1997 definition. Further, in countries where resources are limited, laboratory facilities for detection of dengue viral load and anti-dengue IgG antibodies are often lacking, and the method used to determine and select optimal combining weights to the prune algorithm contained in the decision tree might bias the study results [30].

Several scoring systems have been developed to identify patients at highest risk for DSS. The dengue infection severity score described by Pongpan et al. showed that age >6 years, hepatomegaly, hematocrit ≥40%, systolic pressure <90 mmHg, WBC >5.0×10^9^ cells/L, and platelet counts ≤50×10^9^ cells/L were clinical predictors of DHF/DSS in Thai children [31]. Huy et al., in a study of 444 children with DSS, developed a simple score containing five determinants (shorter admission day, purpura/ecchymosis, ascites/pleural effusion, blood platelet count, and pulse pressure) to predict recurrent shock in dengue [32]. In a study of 1207 children with DSS, the variables in the final scoring model for profound DSS included younger age, earlier day of illness at shock, higher temperature, faster pulse rate, higher hematocrit, and worse hemodynamic status in females [33]. However, similarly, the severity of dengue was defined by the WHO 1997 classification, and application of these criteria commonly does not detect all SD manifestations [8, 9]. Indeed, it is difficult to compare our present results with those of studies dealing with the prediction of DHF/DSS according to the WHO 1997 classification, as the 2009 WHO definition was applied in the current study. Further, hematocrit measurements can be somewhat insensitive, especially if the patient is receiving intravenous fluid therapy, and are also limited by the fact that an individual’s baseline hematocrit value is rarely known [34]. Finally, it should be noted that the study population in the above-mentioned study was limited to children, and did not include adult patients.

Clinically, patients with dengue are often hospitalized for close monitoring due to the lack of a simple reliable clinical tool to distinguish SD from non-SD. In this large cohort of adult patients hospitalized for dengue, 55 SD cases, based on the WHO 2009 criteria (23 of which were also 1997 WHO-defined DSS), were included, and clinical data before progression to SD were analyzed. Given that dengue infection is a dynamic disease that can result in a wide range of manifestations, two scoring algorithms were proposed based on the time after onset of dengue illness. In the febrile phase (dengue illness ≤4 days), we identified four (old age, minor gastrointestinal bleeding, leukocytosis, and platelet count ≥100 × 10^9^ cells/L) significant independent predictors for SD in the derivation cohort. By rounding the regression coefficients into integers, we developed a simple SD risk score (model 1), which was found to be highly predictive of the risk for SD (AUC, 0.848). During the first 4 days of dengue illness, our analysis using a cutoff value of 1 point of the SD risk score (ranging from −2 to 6 points) showed satisfactory sensitivity and specificity for predicting the risk of progression to SD in both the derivation and validation cohorts. Moreover, we also developed a simple SD risk score (model 2) (old age and leukocytosis; AUC, 0.859) that could identify patients with dengue as having SD after day 4 from illness onset. In the derivation cohort, model 2, using a combination of these 2 parameters and a risk score (ranging from 0 to 3 points) cutoff of 1 point, identified SD correctly, with a sensitivity of 80.3%. Despite model 2 showing a high AUC in the validation data, the small sample size in the validation cohort resulted in the difference being statistically insignificant.

The warning signs proposed by the WHO 2009 are considered potential key factors for early recognition of SD; however, the sensitivity of each sign in predicting SD is reportedly poor [10]. In a study of 1507 dengue patients, the sensitivities of the warning signs for predicting DHF and SD were as follows: abdominal pain, 29% and 21%; persistent vomiting, 6% and 8%; hepatomegaly, 1% and 0%; hematocrit rise and rapid platelet count drop, 9% and 5%; clinical fluid accumulation, 21% and 17%; mucosal bleeding, 42% and 17%; and lethargy, 33% and 34%, respectively [10]. The presence of any of these seven warning signs had a sensitivity of 95% but only 18% specificity for SD [10]. In our study, excluding hepatomegaly and lethargy, the incidence of each warning sign before progression to SD was extremely low (Table 3). Our report, along with previous studies [10], indicate that individual warning signs are not appropriate predictors for the early identification of patients at risk of SD. Conversely, the simplified risk score strata established herein correlated strongly with the SD rates in both the derivation and validation cohorts. Clinicians should concentrate efforts on patients with SD risk scores ≥2 points, who are at >50% risk of developing SD (Fig 2), irrespective of the day of illness onset. In contrast, patients with a score <1 point have a comparatively low (<3%) risk of SD (Fig 2). Despite the high predictive value of the score, misclassification (score <1 point) occurred in 14 patients with SD (11 in model 1 and 3 in model) (Fig 2). The current predictive tools identify the majority (75%)—but not all—patients with SD who required aggressive intervention. Notably, the clinical and laboratory features of dengue vary greatly throughout the entire illness [1–5]. Therefore, the implementable predictive tools developed in this study cannot totally replace good clinical judgement for the complexity of individual cases. This information must be acknowledged and addressed so that it can be used effectively and without compromising patient care. Nevertheless, our SD risk score systems have the benefit of being simple to use; each of the risk factors is easily available at presentation or soon afterward. Hence, our study may have important clinical implications on how to allocate the resources, particularly in countries where medical resources are sparse and the burden of dengue is high.

Herein, we showed that old age (≥ 65 years) was significantly associated with SD outcome, similar to in other studies [4, 35]. Of note, all four dengue virus serotypes circulate in Taiwan, and have caused annual epidemics in the same geographical areas of Taiwan over the past 3 decades [3–5]. Secondary dengue infection was previously reported to be a risk factor for the development of DHF/DSS [36, 37]. Thus, in our study cohort, it is likely that elderly patients more frequently presented with secondary dengue infections, increasing their risk of SD. Moreover, elderly patients have worse outcomes compared to younger patients, with increased rates of bacterial co-infection related to morbidity and mortality [5, 35].

Leukocytosis, with or without left shift, is a common result of the infectious process, but can also occur in a wide variety of conditions [5, 38]. Most dengue patients experience leukopenia from bone marrow suppression [39, 40]. A previous study revealed that a WBC >5.0×10^9^ cells/L is a prognostic factor for dengue severity [31], and Nelson et al. revealed that, for DHF patients, 67% and 66% of shock and mortality cases, respectively, had leukocytosis [41]. In our cohort, leukocytosis was found in 13 SD patients at hospital presentation. Further, bacteremia was detected in two patients, and severe gastrointestinal bleeding occurred in 7 after their hospitalization. Leukocytosis is not uncommon in the presence of bacterial co-infection in dengue patients [42, 43]. Nevertheless, leukocytosis can also occur in dengue patients with gastrointestinal bleeding as a consequence of stressful stimuli, and appears to reflect the severity of bleeding [11, 44]. Clinicians need to be aware of the potential for bacterial co-infection and gastrointestinal bleeding in dengue patients with leukocytosis, as they are associated with a greater mortality rate if left untreated.

Mucosal bleeding severity can range from mild local bleeding to severe and potentially fatal hemorrhage. The gastrointestinal tract is the most common site of serious bleeding in dengue, manifested by hematemesis, melena, and hematochezia [11, 45, 46]. In a previous report of 30 fatal dengue cases, 80% of the patients experienced gastrointestinal bleeding [12]. In a study of 11 dengue deaths in Puerto Rico, Tomashek et al. noted that gastrointestinal bleeding occurred in 45% of the cases [47]. Remarkably, during the early stage of dengue illness, minor gastrointestinal bleeding was significantly more prominent among those classified as having SD in our series. Conversely, there was no significant difference in the occurrence of minor gastrointestinal bleeding between SD and non-SD after day 4 from symptoms onset. As reported previously [11, 45, 46], most cases of gastrointestinal bleeding occur on the fourth day after onset of illness, and this was also seen in our dengue patients. It should be noted that in the current study, information on the use of non-steroidal anti-inflammatory drugs and aspirin, which might increase the risk of bleeding, was not obtained [19]. Moreover, we did not detect any severe gastrointestinal bleeding in non-SD cases during the hospitalization, whereas severe gastrointestinal bleeding most frequently occurred in SD patients at the late of the clinical course of dengue illness. In addition, patients with SD had more severe thrombocytopenia compared to the non-SD patients at the early stage of illness (≤ 4 days) in the current series (median platelet count, 39 ×10^9^ cells/L vs. 108 ×10^9^ cells/L; Table 3). Despite thrombocytopenia being common in both DF and DHF [2], studies have shown that the platelet count is inversely correlated with disease severity [48, 49]. Patients with higher severity of thrombocytopenia have a greater risk of bleeding, as well as worse clinical outcomes [48, 49]. Our data highlight the fact that the occurrence of minor gastrointestinal bleeding and a platelet count <50×10^9^ cells/L during the first 4 days of illness are associated with a higher risk of SD.

Previous reports disclosed that diabetes contribute to development of DHF [50, 51]. Our analysis showed that SD patients were significantly more likely to have any comorbidity, but this variable was not significant in the multivariate model. Comorbidity was associated with old age. In the present study, more than 60% (34 of 55 SD patients) of the SD patients were older than 65 years of age. The effect of comorbidity on poor dengue outcomes may be confounded by ageing. Therefore, although more studies are needed, our series revealed that a biological change with the aging process is one of the main factors in the development of SD, and comorbidity may merely be a confounder partly reflecting the aging process [52].

Skin manifestations in dengue range 46–68% [1, 2]. In a school-based prospective open cohort study with a 9,448 person-year follow-up in children, the prevalence of skin rash did not differ between DF and DHF [53]. In our study, we did not find significant association between rashes and dengue severity in the multivariate model. The presence of skin rash could assist clinical suspicious of dengue infection among febrile patients but was unable to predict the clinical outcome.

There are some limitations to our study. Our cohort comprised only adult patients, lacked primary and secondary dengue infection data, and was a retrospective cohort study. Thus, future prospective studies are necessary to validate our findings in different populations for better generalization. Despite these limitations, this study also has several strengths. We defined a simple, practical, and relatively accurate clinical risk score that can be used for early prediction of SD based on different phases of dengue illness. Furthermore, this easily implementable tool may be beneficial in resource-limited settings.

In conclusion, our simple SD risk score can assist clinicians in deciding which dengue patients need hospitalization, and may thereby improve clinical practice by decreasing the number of unnecessary hospitalizations and by reducing mortality and morbidity, particularly in resource-limited countries. It is important to have confirmed dengue cases before this clinical risk score can be applied. Notably, we did not study the utility of SD risk score as diagnostic criteria for probable dengue as all our patients were laboratory confirmed dengue. Patients with bacterial infection or other febrile illnesses may have SD risk score of >1. In accordance with this consideration, the importance of early differentiating dengue from other febrile illnesses, which has been addressed elsewhere [54, 55], should be emphasized so that appropriate monitoring and management can be delivered in a timely fashion in case of high risk for SD.

## Supporting Information

S1 Table Clinical data of 55 severe dengue patients during the entire clinical course of dengue illness. (PDF)
