# Supplementary material for: Development of a Simple Clinical Risk Score for Early Prediction of Severe Dengue in Adult Patients
Source: PLoS One. 2016 May 3;11(5):e0154772. doi: 10.1371/journal.pone.0154772 (PMC4854400; doi:10.1371/journal.pone.0154772)
Supplement: S1 Table — (PDF) [file pone.0154772.s001.pdf]

S1 Table. Symptoms/signs and laboratory features of 55 severe dengue patients during the entire clinical course of dengue illness.

Variable	Severe dengue (n = 55)
**Warning signs**	
Abdominal pain, no. (%)	25 (45.5)
Vomiting, no. (%)	25 (45.5)
Mucosal bleeding, no. (%)	
Gastrointestinal bleeding	32 (58.2)
Hemoptysis	2 (3.6)
Gum bleeding	5 (9.1)
Clinical fluid accumulation, no./No. (%)	
Pleural effusion	27/50 (54)
Ascites	11/38 (28.9)
**Other symptoms/signs, no. (%)**	
Fever	51 (92.7)
Orbital pain	4 (7.3)
Bone pain	24 (43.6)
Myalgia	27 (49.1)
Headache	20 (36.4)

Diarrhea	10 (18.2)
Petechial	19 (34.5)
Cough	19 (34.5)
Rash	9 (16.4)
**Laboratory features**	
Leukocytosis (WBC >10 × 10^9 cells/L), no. (%)	15 (27.3)
Median hematocrit % (range)	
Male	37.1 (22.3–50.4)
Female	35.9 (21.9–49.9)
Severity of thrombocytopenia, no. (%)	
Platelet count >150 × 10^9 cells/L	1 (1.8)
Platelet count 100–149 × 10^9 cells/L	0
Platelet count 50–99 × 10^9 cells/L	5 (9.1)
Platelet count <50 × 10^9 cells/L	49 (89.1)
AST > 400 U/L (normal value < 40 U/L), no./No. (%)	19/49 (38.7)
ALT > 400 U/L (normal value < 40 U/L), no./No. (%)	12/49 (24.5)

ALT = alanine aminotransferase; AST = aspartate aminotransferase; no./No. = number of cases/number of overall cases with data available for evaluation; WBC = white blood cell count.
